# Efficacy of a competitive exclusion culture against extended-spectrum β-lactamase-producing *Escherichia coli* strains in broilers using a seeder bird model

**DOI:** 10.1186/s12917-020-02370-y

**Published:** 2020-05-19

**Authors:** Ulrich Methner, Uwe Rösler

**Affiliations:** 1 Institute of Bacterial Infections and Zoonoses at the Friedrich-Loeffler-Institute, Federal Research Institute for Animal Health, Naumburger Str. 96a, D-07743 Jena, Germany; 2 Department of Veterinary Medicine, Institute of Animal Hygiene and Environmental Health, Free University Berlin, Robert-von Ostertag-Str. 7-13, D-14163 Berlin, Germany

**Keywords:** Competitive exclusion, Broiler chicken, Extended-spectrum β-lactamases, Efficacy, seeder bird model

## Abstract

**Background:**

Administration of a competitive exclusion culture (CE culture) has the potential to induce protective effects in very young chicks against caecal colonisation by EEC (= extended-spectrum β-lactamases [ESBL] and AmpC-type [AmpC] beta-lactamases producing *Escherichia coli*). The study aimed to verify the protective capacity of a CE culture in broilers using the seeder bird model against EEC exposure of the chicks.

**Results:**

Introduction of infected seeder birds resulted in rapid and strong caecal colonisation of four different EEC challenge strains tested in untreated contact broilers. Compared to controls the broilers pre-treated with the CE culture showed a considerable decrease in caecal load of different EEC challenge strains from about 3.0–3.5 log_10_ units (*P* < 0.05) on day 9 of life to 2.5–3.0 log_10_ units (*P* < 0.05) on day 37. A slightly higher protective level of the CE culture in layer birds than in broilers raises the question on reasons for possible differences in the efficacy of CE culture in broiler and layer breeds. Whether the diet’s protein content has an impact on both normal intestinal flora composition and the efficacy of CE cultures against EEC or other pathogens remains open and needs further elucidation.

**Conclusions:**

Our findings suggest that CE cultures of undefined composition can be valuable to reduce the intestinal colonisation by EEC in newly hatched broilers.

## Background

The high prevalence worldwide of EEC (= extended-spectrum β-lactamases [ESBL] and AmpC-type [AmpC] beta-lactamases producing *Escherichia coli*) in the broiler production [1–7] represents an increasing problem for both public health and veterinary medicine. The capability of EEC to enzymatically deactivate beta-lactam antibiotics [8] confers resistance against a variety of antibiotics, such as 3rd and 4th generation cephalosporins, which are listed as “critically important antimicrobials” [9]. Broiler chickens can act as reservoirs of EEC and introduce them into the food production process [2, 4, 10–12]. Compared to other foodstuffs, poultry carcasses and chicken meat are highly contaminated with EEC [3, 6, 13–17] thus increasing exposure to EEC of the human population [18]. Despite the high EEC load of poultry products, more information on their epidemiological role are needed as EEC genotypes detected in chicken meat and humans may differ considerably [6, 10, 13, 17, 19]. In view of the risks involved, measures to reduce the prevalence of EEC in the entire poultry production chain are essential [20, 21]. Effective control strategies must include the breeding and hatchery sector [1, 22] as well as primary broiler production [23–25] and slaughter processes [26, 27] in order to prevent EEC organisms from entering the food chain. EEC contamination at broiler farms is considered the most important source for both primary exposure of newly hatched chicks and horizontal dissemination within the flock [2, 4, 23, 28]. Therefore, in addition to effective hygiene regimes, an early administration of a competitive exclusion culture (CE culture) may be valuable to relevant prevent intestinal EEC colonisation of the highly susceptible newly hatched birds [29–31]. Following basic studies involving White Leghorn chickens with direct crop administration of EEC challenge strains [30], the aim of this study was to verify the protective capacity of a CE culture in broilers using the seeder bird model for EEC exposure of the chicks.

## Results

### Efficacy of a CE culture against intestinal colonisation and systemic invasion of EEC strains in white Leghorn and Ross 308 broiler chickens using a seeder bird model for challenge

The chickens used in this study were shown to be free of EEC strains after hatching. In experiment 1, introduction of 4 White Leghorn seeder birds infected with EEC 1478 N (group 2) and 1479 N (group 4) into a group of 20 untreated control birds resulted in rapid and very high-level caecal colonisation of both EEC challenge organisms in the untreated contact birds (Table 1). Colonisation rates of more than 10^8^ cfu/g caecal contents were reached already on day 7 post exposure and maintained until day 37 of life of the birds. Pretreatment of the contact chicks with the CE culture (groups 1 and 3) resulted in a reduced (*P* < 0.05) caecal colonisation of the EEC challenge strains at all days of examination until the end of the experiment (Table 1) compared to untreated controls. The difference between pre-treated and untreated White Leghorn contact chickens increased from about 2.5–3.0 log_10_ units on day 9 of life to 5.0–6.0 log_10_ units at the end of the trial on day 37 of life the chickens. Differences among challenge strains concerning the intestinal exclusion effect by the CE culture were not detected. The EEC organisms were not isolated from liver of the White Leghorn birds either from untreated or from pre-treated groups throughout the whole experiment (data not shown).

**Table 1:** Number of ESBL/ AmpC *Escherichia coli* (EEC) challenge organisms (mean log_10_ cfu/g of 4 birds) in caecal contents of specific pathogen free White Leghorn chickens pre-treated with a competitive exclusion (CE) culture on day 1 of life or untreated controls exposed to seeder birds infected with EEC 1478 N (SHV-12) or EEC 1479 N (CMY-2) at 2 days of life (5 contact birds/ 1 seeder bird) (experiment 1)

Day of life	Group 1	Group 2	Group 3	Group 4
1	20 contact birds pre-treated with a CE culture	20 contact birds untreated	20 contact birds pre-treated with a CE culture	20 contact birds untreated
2	Addition of 4 seeder birds infected with EEC 1478 N (10^5^ cfu/ bird)	Addition of 4 seeder birds infected with EEC 1478 N (10^5^ cfu/ bird)	Addition of 4 seeder birds infected with EEC 1479 N (10^5^ cfu/ bird)	Addition of 4 seeder birds infected with EEC 1479 N (10^5^ cfu/ bird)
	Caecal contents	Caecal contents	Caecal contents	Caecal contents
9	6.2^a^	8.7	5.7^b^	8.8
16	5.7^a^	8.5	4.0^b^	8.6
23	3.2^a^	8.3	3.0^b^	8.6
30	3.0^a^	8.2	2.9^b^	8.6
37	2.2^a^	7.8	2.4^b^	8.5

^a^significantly lower than group 2; ^b^significantly lower than group 4

Standard error: 0.374

Using the same experimental design, 4 Ross 308 broiler seeder birds (experiment 2) infected either with EEC 1478 N or 1479 N were placed each in a group of pre-treated or untreated contact broiler chickens on day 2 of life of the birds (Table 2). Rapid multiplication of both EEC challenge strains was also observed in untreated contact broilers, however, the level of caecal colonisation was about 2.0 log_10_ units lower compared with White Leghorn birds in experiment 1. The broilers pre-treated with the CE culture showed a reduction in caecal load of the challenge strains compared with controls from about 3.0–3.5 log_10_ units on day 9 of life to 2.5–3.5 log_10_ units (both significantly, *P* < 0.05) on day 37 of life the broilers. The total caecal count of EEC challenge strains 1478 N and 1479 N in pre-treated broilers (Table 2) was, apart from day 9 of life, very similar to that detected in pre-treated White leghorn birds (Table 1). However, the difference between treated and untreated broilers amounted to only 2.0–3.5 log_10_ units during the whole experiment, whereas the difference in White Leghorn chicks (Table 1) was about 5.0–6.0 log_10_ units from day 23 of life until the end of the experiment. As seen in experiment 1 the EEC challenge strains were not detected in liver of the broilers neither in untreated nor in pre-treated chicks throughout the whole experiment (data not shown). To better verify the results obtained in broilers, experiment 3 was carried out under identical conditions using further EEC strains for challenge. Four Ross 308 broiler seeder birds infected with EEC 1475 N or 1476 N were placed each in a group of pre-treated or untreated contact broiler chickens on day 2 of life of the birds (Table 3). A rapid and strong caecal colonisation of both EEC challenge organisms was observed in the untreated contact birds, which was higher (approx. 1.0–2.0 log_10_ units) than in experiment 2. Pretreatment of the contact chicks with the CE culture (groups 1 and 3) resulted in a reduced (*P* < 0.05) caecal colonisation of the EEC challenge strains at all days of examination until the end of the experiment (Table 3) compared to the corresponding untreated controls. Despite the statistically significant (*P* < 0.05) difference between treated and untreated chickens amounted also in this broiler experiment to not more than 2.0–2.5 log_10_ units between days 16 and 37 of life. Also in this experiment, the EEC challenge strains were not detected in liver of the broilers neither in untreated nor in pre-treated chicks throughout the whole trial (data not shown). The observed considerable increase in the difference in caecal counts of EEC challenge organisms between pre-treated and untreated White Leghorn birds from day 9 to day 37 of life (experiment 1) was not seen in Ross 308 broiler chickens (experiments 2 and 3).

**Table 2:** Number of ESBL/ AmpC *Escherichia coli* (EEC) challenge organisms (mean log_10_ cfu/g of 4 birds) in caecal contents of broiler chickens pre-treated with a competitive exclusion (CE) culture on day 1 of life or untreated controls exposed to seeder birds infected with EEC 1478 N (SHV-12) or EEC 1479 N (CMY-2) at 2 days of life (5 contact birds/ 1 seeder bird) (experiment 2)

Day of life	Group 1	Group 2	Group 3	Group 4
1	20 contact birds pre-treated with a CE culture	20 contact birds untreated	20 contact birds pre-treated with a CE culture	20 contact birds untreated
2	Addition of 4 seeder birds infected with EEC 1478 N (10^5^ cfu/ bird)	Addition of 4 seeder birds infected with EEC 1478 N (10^5^ cfu/ bird)	Addition of 4 seeder birds infected with EEC 1479 N (10^5^ cfu/ bird)	Addition of 4 seeder birds infected with EEC 1479 N (10^5^ cfu/ bird)
	Caecal contents	Caecal contents	Caecal contents	Caecal contents
9	2.3^a^	6.6	3.2^b^	6.1
16	4.4^a^	6.3	4.9^b^	6.5
23	3.5^a^	6.5	3.0^b^	6.6
30	2.8^a^	5.5	2.8^b^	6.4
37	2.4^a^	5.9	3.3^b^	5.9

^a^significantly lower than group 2; ^b^significantly lower than group 4

Standard error: 0.487

**Table 3:** Number of ESBL/ AmpC *Escherichia coli* (EEC) challenge organisms (mean log_10_ cfu/g of 4 birds) in caecal contents of broiler chickens pre-treated with a competitive exclusion (CE) culture on day 1 of life or untreated controls exposed to seeder birds infected with EEC 1475 N (CTX-M-15) or EEC 1476 N (TEM 20) at 2 days of life (5 contact birds/ 1 seeder bird) (experiment 3)

Day of life	Group 1	Group 2	Group 3	Group 4
1	20 contact birds pre-treated with a CE culture	20 contact birds untreated	20 contact birds pre-treated with a CE culture	20 contact birds untreated
2	Addition of 4 seeder birds infected with EEC 1475 N (10^5^ cfu/ bird)	Addition of 4 seeder birds infected with EEC 1475 N (10^5^ cfu/ bird)	Addition of 4 seeder birds infected with EEC 1476 N (10^5^ cfu/ bird)	Addition of 4 seeder birds infected with EEC 1476 N (10^5^ cfu/ bird)
	Caecal contents	Caecal contents	Caecal contents	Caecal contents
9	2.8^a^	7.2	5.6^b^	8.6
16	5.3^a^	7.9	6.6^b^	9.1
23	6.1	7.3	6.8^b^	8.5
30	5.9^a^	7.6	6.1^b^	7.8
37	3.7^a^	6.1	5.7^b^	7.8

^a^significantly lower than group 2; ^b^significantly lower than group 4

Standard error: 0.405

## Discussion

In view of recent data on the occurrence of EEC in the poultry meat production chain [1, 2, 14, 24, 26, 27, 32] an effective control of EEC organisms requires a top-down approach to include all stages from breeding stock, hatcheries, broiler production, slaughter and processing up to kitchen hygiene. Apart from vertical EEC transmission to meat-producing broilers [20–22], horizontal transmission [4, 23, 28, 33] due to contaminated environment of broiler production facilities are considered as crucial for exposure of newly hatched broiler birds. Persistence, circulation and survival of EEC at farms despite cleaning and disinfection procedures, may result in rapid intestinal colonisation and high prevalence rates at broiler production flocks shortly after entry. Effective control at farm level should include both i) the reduction of the environmental EEC load through effective hygienic regimes to minimise exposure of the birds quantitatively and ii) the increase of resistance of the newly hatched broilers against EEC. Gut flora preparations administered to chicks shortly after hatching proved their efficacy not only against a large variety of *Salmonella* serovars [34] but also against EEC [29, 31]. Basic studies using White Leghorn chickens with crop instillation of the EEC challenge organisms [30] revealed considerable but varying protective effects of a CE culture against individual EEC strains. To verify conditions closer to the field, both broiler chickens and the seeder bird model for EEC exposure were used to further explore CE culture efficacy. To enable comparison with results from our previous study [30], we used the same strong colonising strains EEC 1478 N and EEC 1479 N for infection of both White Leghorn and Ross 308 broiler chickens in seeder bird experiments 1 and 2. The selected design of the seeder bird model [35] enabled rapid EEC dissemination in untreated White leghorn and broiler breeds. However, despite using the same EEC challenge strains and the substantial protection provided by the CE culture, caecal EEC count differences between CE-treated and untreated broilers amounted to 2.0–3.5 log_10_ units of reduction during the entire experiment, whereas the difference in White leghorn chicks was about 5.0–6.0 log_10_ units from week 3 of life until the end of the experiment. The considerable protective effect by a CE culture in broilers was confirmed in another experiment where additional EEC genotypes were used for infection. However, the difference in the intestinal count of challenge organisms between CE-inoculated groups and controls again amounted to not more than 2.0–2.5 log_10_ units from day 16 until day 37 of life. Furthermore, the increasing difference between CE-treated and untreated birds observed in the layers between days 9 and 37 of age was not seen in the experiments involving the broiler breed. Although comparison of data from different trials is not justified, observations from the present study raise the question whether there are reasons for possible differences in the efficacy of a CE culture between broiler and layer breeds. One of the most important differences between broiler and layer breeds, which affect both natural and administered intestinal microfloras is the composition and the amount of feed ingested. The considerably higher proportion of protein in the broiler diet [36] compared to layers may result in a different composition of the intestinal flora. For instance, it was shown that the dietary protein content affected diversity and population number of microbiota resulting in a modulation of the *Campylobacter jejuni* pathogenesis in broilers [37]. A higher protein content in feed was associated with a higher caecal *Campylobacter jejuni* load [38], therefore, reducing the availability of protein could limit the spread of infection [36]. Infection studies between broiler-and layer-type chickens [37] provided evidence that the colonisation pattern of commensal bacteria may depend on the breed and/ or the corresponding diet. Furthermore, the CE culture used in this study originated from SPF chickens (Product information) and not from broilers, therefore, the intestinal colonisation of its microbiota in layer or broiler breeds as well as the protective effects induced in the breeds may be different. From the results of this study, differences in the efficacy of a CE culture between layer or broiler breeds can neither be confirmed or precluded, further research is needed to comprehensively verify the impact of the diet on the protective potential of CE cultures against EEC and other pathogens in different breeds.

Apart from these so far neglected aspects, some more practical factors are essential to exploit the protective potential of gut flora preparations. Administration of the CE culture prior to the first uptake of the pathogen is the indispensable prerequisite for any efficacy of a CE culture [29, 31, 34, 39]. Therefore, the chickens must be free of EEC after hatching, which highlights the need for effective control already in the breeder sector. Despite the observed differences of a commercial CE culture against individual EEC strains [30], the protective effect in layer and broiler breeds was evident in the present study. However, the undefined composition of CE cultures precludes their registration in many countries. The development of reproducibly effective gut flora preparations of defined composition against EEC that are acceptable for registration is a highly challenging and time-consuming undertaking. Therefore, in view of the high prevalence of ESBL-producing *Enterobacteriaceae* in poultry and the proven efficacy of CE cultures, these preparations to register not as medicinal products or vaccines, but under the novel product category of “normal gut flora” has been proposed [40].

## Conclusions

Using the seeder bird model for exposure, a commercial CE culture has been shown to substantially reduce the caecal colonisation of extended-spectrum β-lactamases and AmpC-producing *Escherichia coli* strains in broilers, thus confirming its efficacy as a tool for EEC control. A higher protective level of the CE culture in layer birds compared with broilers raises the question on possible reasons for differences in the efficacy of a CE culture in broiler and layer breeds. Whether the dietary protein content may impact the efficacy of the CE culture to limit the colonisation of EEC or other pathogens in broilers and layers remains open, but might be further elucidated.

## Methods

The methodology used in this study has partly been published previously [30] to compare and extend results.

### Chickens

Specific pathogen-free White Leghorn chickens (Valo BioMedia GmbH, Germany) and Ross 308 broiler chicks (Aviagen Ltd., The Netherlands) were hatched at the facilities of the Friedrich-Loeffler-Institute. After hatching, samples from the incubators (faeces, dust) were collected and examined for the occurrence of EEC exactly as described [20, 30]. Experimental and control groups were kept in floor pens according to the German regulation [41] in separate negative pressure rooms. Each room had its own anteroom where the personnel changed clothes and shoes before entering. White leghorn chickens received commercial layer-rearing feed (coarse meal without antibiotics), Ross 308 broiler chickens were fed with commercial starter (days 0–10), grower (days 11–24) and finisher broiler feed (days > 25) without antibiotics (Aviagen Ltd); feed and public drinking water were available ad libitum during all animal trials. All rooms, anterooms and equipment were cleaned and disinfected before each experiment. After disinfection, surfaces in rooms and on equipment were swabbed and tested for the absence of EEC. Cleaning and feeding regimes were arranged to effectively prevent cross-contamination throughout the experiments. Animal experiments were performed in accordance with the German Animal Protection Act and approved by the animal-welfare body of the state office for consumer protection in the German federal state Thuringia (registration number: 04–002/16) as stated [30].

### Bacterial strains and culture

A commercially available CE culture (Aviguard, Microbial Developments Ltd.) was used to pretreat the birds on day 1 of age after dissolving and applied via crop instillation [30]. Four EEC strains from healthy broilers [28, 33] with different ESBL-genes (EEC 1475 N - blaCTX-M15; EEC 1476 N - blaTEM-52; EEC 1478 N - blaSHV-12) or an AmpC-gene (EEC 1479 N-blaCMY-2) were selected for exposure of the birds because of their colonising properties [30]. The antimicrobial susceptibilities of the strains were valued by estimating the minimum inhibitory concentration (MIC) exactly as described [30, 42]. The EEC organisms selected for infection were resistant against sulfamethoxazole, trimethoprim, ciprofloxacin, tetracycline, nalidixic acid, cefotaxime, tigecycline, ceftazidime and ampicillin [30]. Oral administration of seeder birds with the EEC strains was carried out with doses of 2 × 10^5^ cfu/ bird via crop instillation in all experiments. Doses for infection were adjusted as described, all strains had been stored in a Cryobank system (Mast Diagnostica) at − 20 °C [30].

### Experimental design and bacteriology

In experiment 1, two groups of 20 White Leghorn contact birds each were pre-treated on day 1 of life with the CE culture (Table 1). On day 2 of life, the chicks of these groups and two control groups were exposed each to 4 seeder birds infected with either EEC 1478 N (groups 1 and 2) or EEC 1479 N (groups 3 and 4) at a dose of 1–2 × 10^5^ cfu per bird on day 1 of age. In experiment 2, two groups of 20 Ross 308 broiler contact chickens each were pre-treated on day 1 of life with the CE culture (Table 2). On day 2 of life, the birds of these groups and two control groups were exposed each to 4 seeder broiler birds administered with either EEC 1478 N (groups 1 and 2) or EEC 1479 N (groups 3 and 4) at a dose of 1–2 × 10^5^ cfu per bird on day 1 of age. In experiment 3, two groups of 20 Ross 308 broiler chicks each were pre-treated on day 1 of life with the CE culture (Table 2). On day 2 of life, the birds of these groups and two control groups were exposed each to 4 seeder broiler birds infected with either EEC 1475 N (groups 1 and 2) or EEC 1476 N (groups 3 and 4) at a dose of 1–2 × 10^5^ cfu per bird on day 1 of age.

In all experiments, the EEC challenge strains were quantified in caecal contents (Tables 1, 2 and 3) and livers (data not shown) of 4 contact birds/ group at days 9, 16, 23, 30 and 37 of life by a standard plating method [30, 39, 43] after stunning and killing of the chickens [30, 44, 45]. Criterion to evaluate the efficacy of the CE culture is the number of challenge organisms in caecal contents in pretreated compared with control groups. This difference is termed as protective effect induced by the CE culture. To ensure a ratio of 5:1 between contact and seeder birds during the whole experiment one seeder bird each was taken out of the groups on days 16, 23, 30 and 37 of life in all trials (data not shown). Stunning of the birds was carried by percussive blow to the head [44], killing by decapitation [45]. Homogenised organ samples were diluted in phosphate buffered saline and plated on MacConkey agar (SIFIN) with sodium nalidixate (N) and incubated at 37 °C for 18–24 h [30]. Additionally, caecal contents and liver samples were pre-enriched in buffered peptone water (SIFIN, Germany), incubated at 37 °C for 18–24 h and streaked onto MacConkey agar with sodium nalidixate (SIFIN, Germany) as described [30].

### Statistical analysis

Viable bacterial counts were transformed into logarithmic form. For statistical purposes a viable count of log_10_ < 1.47 (the limit for direct plate detection) from a sample proven to be positive only after enrichment was rated as log_10_ = 1.0. A sample which demonstrated no EEC growth after enrichment was rated as log_10_ = 0. Data were calculated using multifactorial variance analysis followed by “Multiple Range Test”. The factors taken into account were group and time. *P* values < 0.05 were regarded as statistically significant (software: statgraphics plus, Inc. Rockville, MD).

## Data Availability

Data generated or analysed during this study are included in this published article, further information are available from the corresponding author on reasonable request.

## Abbreviations

CE: Competitive Exclusion
EEC: Extended-spectrum β-lactamases [ESBL] and AmpC-type [AmpC] beta-lactamases producing *Escherichia coli*
EUCAST: European Committee on Antimicrobial Susceptibility Testing
MIC: Minimum inhibitory concentration
